# Understanding barriers to effective management of influenza outbreaks by residential aged care facilities

**DOI:** 10.1111/ajag.12595

**Published:** 2018-12-10

**Authors:** Essi Huhtinen, Emma Quinn, Isabel Hess, Zeina Najjar, Leena Gupta

**Affiliations:** ^1^ Sydney Local Health District Public Health Unit Sydney New South Wales Australia; ^2^ School of Public Health Sydney Medical School University of Sydney Sydney New South Wales Australia

**Keywords:** disease outbreaks, infection control, influenza, human, surveys and questionnaires, vaccination

## Abstract

**Objective:**

To identify the perceived barriers to the implementation of the Australian national guidelines on influenza outbreak management with Sydney Local Health District (SLHD) residential aged care facility (RACF) staff.

**Methods:**

All SLHD RACFs were invited to participate in a telephone interview. The questionnaire collected information about demographic characteristics and participants' level of agreement with statements regarding perceived barriers to implementing the national guidelines for influenza outbreak management.

**Results:**

Twenty‐eight of 61 RACFs (46%) participated in the study. The three most common barriers identified were as follows: scepticism towards staff influenza vaccination (n = 13, 46%); the effort required to read the national guidelines (n = 11, 39%); and lack of infrastructure to physically separate residents during an outbreak (n = 10, 36%).

**Conclusions:**

We recommend implementing and evaluating programmes which address misconceptions about influenza vaccination amongst RACF staff. Further, all RACF staff, including care staff, should receive targeted education on the role of infection control in influenza outbreak management.

**Policy Impact**

Our results highlight that more work is needed to implement and evaluate programs which address misconceptions about influenza vaccination and infection control among residential aged care staff.

**Practice Impact**

Our results highlight that there are knowledge gaps among residential aged care staff members, and targeted education should be provided to improve their knowledge and understanding of influenza outbreak management.

## Introduction

People over 65 years of age in residential aged care facilities (RACFs) are at risk of severe disease and death from influenza due to pre‐existing morbidities 1, lower immunogenicity following vaccination 2, and living in close proximity to one another 3. In New South Wales (NSW), Australia, under the *Public Health Act 2010*, influenza is notifiable by laboratories to public health authorities 4. While the reporting of influenza outbreaks in institutions is not mandatory, the *Guidelines for the Prevention, Control and Public Health Management of Influenza Outbreaks in Residential Care Facilities in Australia* encourage RACFs to report suspected or confirmed influenza outbreaks 3. The number of influenza outbreaks in NSW institutions has increased markedly in recent years, the majority being in RACFs 5, 6. Effective outbreak management relies on timely control measures. There is evidence that RACFs experience difficulties managing influenza outbreaks despite the availability of guidelines to assist with outbreak prevention and management 7, 8. However, there is no reported research regarding the perceived barriers by RACFs to implementing these guidelines. In order to provide more targeted education and support to RACFs to enable facilities to effectively prepare for and manage influenza outbreaks, we conducted a study to identify the perceived barriers to the implementation of the Australian guidelines on influenza outbreak management with RACF staff in an inner city Sydney region.

## Methods

Sydney Local Health District (SLHD) is one of eight local health districts covering the Sydney metropolitan area, and the local public health unit (PHU) receives and manages notifications of influenza outbreaks in RACFs. In May 2016, an email was sent to all SLHD RACFs (*n* = 61) with an information package and a study participation invitation. Facilities were contacted via telephone and asked to nominate a suitable study participant to represent the facility. Study participants were required to be at minimum a registered nurse (RN) with involvement in influenza outbreak management in the RACF. A signed consent form was considered agreement to participate.

Interviews with participants were conducted by telephone, using a semi‐structured questionnaire. The questionnaire collected data on participant demographic characteristics, facility demographic characteristics, participants' level of agreement with statements regarding perceived barriers to implementing the national guidelines for influenza outbreak management (using a Likert response scale), and the availability of outbreak management plans and vaccination policies within their facility. The preparedness checklist in the national guidelines was used to guide the development of the questions 3. Participants were also asked to openly reflect on additional barriers to use of the guidelines.

All data were entered into the Research Electronic Data Capture 9 and extracted for further analysis in Microsoft Excel. The reported additional barriers were analysed independently by two of the authors by categorising all comments into key themes. Ethical approval was granted by SLHD Research Ethics Governance Office (Protocol No X16‐0071 & HREC/16/RPAH/89).

## Results

### Demographics

Twenty‐eight of 61 RACFs (46%) participated in the study. Table 1 outlines the demographic characteristics of participants and their facilities. Half of the participants were RACF managers (*n* = 14, 50%). Most facilities (*n* = 24, 85%) had a staff member responsible for infection control; however, only one respondent reported this to be part of their role. Almost all facilities (*n* = 26, 93%) had an influenza outbreak management plan, and the majority organised an influenza vaccination program for residents (*n* = 27, 96%) and staff (*n* = 25, 89%). The total number of RACF beds in each facility ranged from 41 to 140 (median 66).

**Table 1:** Demographic characteristics of responding residential aged care facilities in Sydney Local Health District (*n* = 28)

Demographic characteristics (*n* = 28)	*n* (%)
Respondents' role in organisation	
Director of nursing	4 (14)
Facility manager	14 (50)
Senior registered nurse	9 (32)
Other: CEO	1 (4)
Facility ownership	
Not‐for‐profit organisation	17 (61)
Privatively owned organisation	11 (39)
Type of care (more than one/facility)	
Independent living	3 (11)
Residential care	26 (93)
Dementia care	20 (71)
Number of residents in the facility	
41–60	11 (39)
61–80	7 (25)
81–100	3 (11)
>100	7 (25)
Residents to care staff member (excluding RNs) ratio in any 24‐hour period in the facility	
3–4:1	21 (75)
5–6:1	3 (11)
7–8:1	3 (11)
>10:1	1 (4)
Residents to RN ratio in any 24‐hour period in the facility	
1–10 residents:1 RN	3 (11)
11–20 residents:1 RN	18 (64)
21–30 residents:1 RN	4 (14)
>30 residents:1 RN	3 (11)
Number of general practitioners servicing the facility	
1–3 GPs	1 (4)
4–6 GPs	8 (29)
7–10 GPs	7 (25)
>10 GPs	12 (43)

RN, registered nurse.

Respondents could choose multiple options in relation to forms of care their RACF provided; the most commonly reported types of care provided were residential care (*n* = 26, 93%) and dementia care (*n* = 20, 71%). In most RACFs, in any 24‐hour period, care staff members (staff with basic nursing training) looked after three to four residents (*n* = 21, 75%) and one RN was responsible for 11–20 residents (*n* = 18, 64%).

### Most commonly and least commonly reported barriers

Participants' level of agreement with statements regarding perceived barriers to effective outbreak management as per the national guidelines is summarised in Table 2. The three most common barriers identified were as follows: (i) scepticism towards staff influenza vaccination (*n* = 13, 46%); (ii) the guidelines taking a lot of time and effort to read (*n* = 11, 39%); and (iii) facilities lacking the physical infrastructure to separate or cohort residents during an outbreak (*n* = 10, 36%).

**Table 2:** Participants' level of agreement with statements about the potential barriers to implanting the national guidelines survey questions, by percentage

Statements	Agree/Strongly agree (%)	Disagree/Strongly disagree (%)	Unsure (%)
Staff are sceptical towards influenza vaccination	46	50	14
The national guidelines take a lot of time and effort to read	39	46	14
Lack of infrastructure to separate cohort residents during an outbreak	36	64	0
There are a large number of competing priorities	32	64	4
There are issues accessing antivirals in a timely manner	32	54	14
There is a lack of a designated outbreak management team with clear roles and responsibilities	22	71	7
There is a lack of a designated person responsible for outbreak prevention and management	11	79	11
The national guidelines are unrealistic to implement	7	79	14
There is a lack of agreement between GPs regarding antiviral use for their respective patients	11	82	7
An influenza outbreak is unlikely to occur in our facility	11	82	7
There is a lack of funding to implement vaccination programs	4	93	4
There is a lack of training programs for outbreak prevention and management	7	93	0
There are a high number of casual or agency staff	7	93	0
There are insufficient resources to implement staff education programs	4	96	0

A number of factors were assessed by participants as not being barriers to effective outbreak management. The most common such factors were as follows: (i) facilities lacking staff education resources (*n* = 26, 96%); (ii) a high number of casual or agency staff (*n* = 26, 93%); (iii) lack of funding for vaccination programs (*n* = 26, 93%); and (iv) lack of training programs for outbreak prevention and management (*n* = 26, 93%).

### Participant's reflections of additional barriers

Responses to additional barriers noted by participants were coded into two main themes (in order of theme strength): (i) observed lack of compliance with infection control recommendations by RACF staff, likely due to lack of understanding and education; and (ii) difficulties with preparing for and implementing medical interventions, for example the high cost of antiviral medication and the need to access this within 24 hours of notification of a confirmed outbreak.

## Discussion

This study provides an important perspective from RACF staff about some of the barriers to preparing for and managing influenza outbreaks in RACFs. Participants reported that RACFs were well resourced to offer staff education programs and have access to outbreak management training programs. Despite this, our findings suggest that there is noncompliance with recommendations pertaining to infection control and outbreak prevention measures. This was most notable in relation to recommendations relating to staff influenza vaccination, with the highest number of respondents agreeing that staff are sceptical towards the vaccine.

Our results provide additional insight into healthcare staff attitudes towards influenza vaccination. We found that most SLHD RACFs already fund coordinated influenza vaccination programs, yet low staff vaccination rates 8 indicate that the majority of staff do not utilise these. Commonly cited reasons given by healthcare workers for not receiving the influenza vaccine are misconceptions regarding the vaccine or lack of availability 10; our study reports staff scepticism towards the vaccine as the main issue. In an effort to increase staff influenza vaccination, in April 2018, the Commonwealth government mandated aged care providers to offer influenza vaccination to all staff. Vaccination remains the best available defence against contracting influenza despite conflicting reports of the efficacy of the vaccine and the perceived side effects 2, 11. However, our findings suggest that providing staff vaccination may only be successful if paired up with education programs addressing influenza vaccine myths. Additional benefits from successful vaccination programs for RACF staff are likely to include a reduction in disease transmission from staff to residents 12, 13, 14 and an improvement on the pandemic preparedness of the facility, as reported in research conducted in the United States, where high staff influenza vaccination rates were positively correlated with pandemic preparedness 15. Therefore, targeted education programs across all aspects of influenza vaccination should be provided to all RACF staff, including those with direct care responsibilities, with the aim to increase staff vaccination rates.

Almost all study participants were satisfied with the educational resources available to RACF staff, in keeping with the National Aged Care Workforce census findings 16. However, the perceived noncompliance with infection control recommendations reported by some participants was attributed to lack of education, indicating a potential gap between education and clinical practice. This observation could be explained by the growing shift in staffing structures within RACFs nationally, with the proportion of care staff (compared to RNs) increasing significantly in recent years. During the last census, care staff constituted 70% of the RACF workforce 16. Care staff are an occupational group with basic nursing training and spend most time providing direct care to residents 16. In comparison, RNs are increasingly involved with managerial duties and have less time to provide direct care 16, 17. Hence, it is vital to ensure that those with direct care responsibilities have access to education which promotes an understanding of the core principles of infection control 18. Currently however, generalised mandatory training is the most common type of training done by RACF staff overall, with mainly the more specialised professional groups partaking in other forms of professional development and training 16. Despite this, few facilities have any nursing staff with a higher certification in infection control 19. Further research is needed to evaluate the actual level of understanding of infection prevention measures amongst all professional groups in RACFs.

This study was limited by a small sample size, and being conducted in only one area in NSW, thereby the generalisability of findings is unclear. We were also unable to assess the demographics of nonresponders to our survey and thus cannot estimate the potential for sampling bias. It is also possible that responding facilities were more likely to be engaged with outbreak management as our recruitment strategy only included RNs, who have access to annual outbreak management training courses run by the PHU.

## Conclusions

Our findings identified potential compliance problems with infection prevention and control strategies, including scepticism towards staff influenza vaccination, despite programs and resources available for staff education. We recommend therefore that staff influenza vaccination strategies include education programs addressing influenza vaccine myths. It is also important to recognise the shift of occupational roles within RACFs and promote relevant infection prevention and control education, targeting especially those with direct care responsibilities. In Australia, those with direct care responsibilities in RACFs have the least access to professional development yet the most exposure to vulnerable people in these settings.
